# Alpha-enolase involvement in intestinal and extraintestinal manifestations of celiac disease

**DOI:** 10.1016/j.jtauto.2021.100109

**Published:** 2021-06-16

**Authors:** Aaron Lerner, Polina Sobolevskaia, Leonid Churilov, Yehuda Shoenfeld

**Affiliations:** a Chaim Sheba Medical Center, The Zabludowicz Research Center for Autoimmune Diseases, Tel Hashomer, Israel; b Saint Petersburg State University, St. Petersburg, Russia; c Sechenov First Moscow State Medical University of the Ministry of Health of the Russian Federation (Visiting Professor), Moscow, Russia; d Ariel University, Ariel, Israel

**Keywords:** Celiac disease, Neuron-specific enolase, Anti-alpha-enolase, Gluten, Extraintestinal manifestation, Autoimmune disease, celiac disease, CD, neuron-specific enolase, NSE, amine precursor uptake and decarboxylation, APUD, gastroenteropancreatic neuroendocrine tumors, GEP-NETs, alpha-enolase, ENO1, anti-enolase antibodies, AAbs, anti-alpha-enolase antibodies anti, AAE Ab, gluten free diet, GFD

## Abstract

Celiac disease is a life-long intestinal autoimmune disease, characterized by the gluten intolerance and chronic enteric inflammation. Traditionally presented by intestinal manifestations, however, a shift toward extra intestinal presentation is taking place. One of the affected organs is the nervous systems presented by neuropsychiatric manifestations, hence the mechanism and pathways are not clear. The presence of neuronal and alpha-enolases and their corresponding antibodies were noticed in the mucosa and serum of celiac disease patients, as well as in other various autoimmune diseases with psycho-neurological manifestations. The aims of the present review are to screen the literature on different isoforms of enolase, mainly alpha enolase, and their specific antibodies and to suggest their potential pathophysiological mechanisms relaying the enolases to intestinal or extraintestinal celiac disease manifestations. The shared aspects between the enolases and celiac disease and the cross-talks between alpha-enolase and tissue transglutaminase suggest new potential pathophysiological mechanisms that might drive celiac disease evolvement.

## Highlights

• Celiac disease is an autoimmune disease affecting mainly the small intestine and also the nervous systems.
• Neuronal and alpha-enolases and their corresponding antibodies are present in celiac patient's mucosa and serum.
• The shared aspects between the enolases and celiac disease might open new therapeutic aspects for celiac disease.

## 1 Introduction

Celiac disease (CD) is a frequent autoimmune entity presented in genetically predisposed people when consuming gluten-containing prolamins (i.e., wheat, rye, barley or oat) or their constituents [1]. Its prevalence is continuously rising, affecting around 1–2% of the Western populations [2,3]. Geo-epidemiological, HLA-DQ2/8 worldwide genotypes distribution, co-localized higher wheat and lower rice consumptions are related to its increase prevalence, thus, attest for an environmental-genetic interplay in CD development [[4], [5], [6]]. In fact, clinically, serologically and pathologically, CD contains several subtypes, spanning potential, latent and classical CDs. The disease is often under or miss diagnosed. The diagnosed/undiagnosed *ratio* is 1/7, respectively [7]. Interestingly, CD phenotype is changing: its incidence, presenting age and symptoms are changing. Seldom are infantile failure to thrive, abdominal pain, bloating and diarrhea the presenting symptoms [4,5,8]. They are replaced by multiple extraintestinal manifestations [[9], [10], [11], [12]], and the brain is included [13]. CD patients tend to have additional autoimmune diseases and their sera contains multiple autoimmune antibodies, including those that target the brain and the peripheral neuronal components [14]. Intriguingly, CD patients can present with central and peripheral neuronal symptoms, with psychiatric and with behavioral manifestations [[15], [16], [17]].

Enolase is a glycolytic enzyme, which can be presented in 3 variants: alpha-enolase, beta-enolase and gamma-enolase. Each isoform is expressed by different gene and their tissue distributions are unique: Alpha-enolase is ubiquitous, beta-enolase is muscle-specific and gamma-enolase is neuron-specific (NSE). The expression of NSE is a useful index of neural maturation because it quite late appearance during neural differentiation. It is accepted as a specific marker for neurons, peripheral neuroendocrine tissue and amine precursor uptake and the decarboxylation (APUD) cells. Moreover, it can act as a biochemical marker for tumors originated from those cells. Interestingly, NSE was detected in all types of neurons like granule cells, Purkinje cells, projection neurons, sensory and autonomic neurons. It is expressed in a plethora of cells like pinealocytes, pituitary glandular and peptide-secreting cells, thyroid parafollicular cells, adrenal medullary chromaffin cells, cells in the islets of Langerhans, Merkel's cells in the skin, neuroendocrine cells in the lung and even in erythrocytes. An increased tissue expression of NSE and increased serum levels of NSE are associated with malignant tissue proliferation [18], and are elevated in patients with gastroenteropancreatic neuroendocrine tumors (GEP-NETs) [19]. Increased level of NSE were also found in Guillain-Barré syndrome [20,21], Creutzfeldt-Jakob disease, meningeal hemorrhage, schizophrenia [21], CD and Crohn's disease [22].

Alpha-enolase (ENO1) - is an enzyme that is involved in diverse metabolic processes including glycolysis, the regulation of cell growth and differentiation and anaerobic metabolism. The upregulation of enolase during metabolic processes as well as the release from dying cells may lead to its uptake by antigen-presenting cells. The subsequent B cell activation triggers an excessive production of anti-enolase antibodies (AAbs) that can potentially initiate tissue injury, e.g., as a result of immune complex deposition [23]. In fact, circulating anti-alpha-enolase antibodies (anti-ENO1 Ab) were identified in numerous autoimmune diseases like autoimmune retinopathy and cancer-associated retinopathy [24,25], ANCA positive vasculitis [26], systemic and multiple sclerosis [27,28], Behçet's disease [29], rheumatoid arthritis patients [30], ulcerative colitis and Crohn's disease [31], lupus nephritis, mixed cryoglobulinemia and primary membranous nephropathy [32,33]. Zooming on the nervous systems, positive anti-ENO1 Ab were demonstrated in autoimmune diseases associated with CNS impairment, such as lymphocytic hypophysitis and Hashimoto's encephalopathy [34,35]. However, it should be stressed that anti-ENO1 Ab were also found in healthy subjects [36]. The role of any isoforms of enolase as well as the role of their corresponding antibodies in nervous system impairments is still unclear. The aims of the current review were to screen the available literature on NSE and AAE Ab in CD and to suggest possible mechanisms and pathways that connect alpha enolase to CD neuropsychiatric manifestations.

## 2 Material and methods

A systematic literature search exploring articles published in PubMed, MEDLINE, LILACS and Scielo dating from 1989 to October 2020, was performed. The search terms were “enolase and nervous system impairments”, “enolase and celiac disease”, “anti-enolase antibodies and celiac disease”, “celiac disease and psychiatric symptoms” and “celiac disease and nervous system”. In total, 43 articles were included in the present review, all eluded to psycho-neurological manifestations of CD, the role of enolase and anti-enolase antibodies in CD as well as its role in psycho-neurological-behavioral manifestations of CD.

## 3 Results

Neuron-specific enolase and CD were first mentioned in 1995 [37]. Biopsy specimens from patients with CD (n = 10), Crohn's disease (n = 13), carcinoma of the duodenum (n = 8) and normal controls (n = 16) were explored for NSE. An increased staining of NSE in the mucosa in CD and Crohn's disease was shown. It was the first time that increased nerve filaments were reported in the mucosa in CD and Crohn's disease. NSE staining was more noticeable in CD than in Crohn's disease. In 2003 a group of scientists from Czech Republic analyzed the sera and intestinal biopsy specimens of patients with CD (n = 21). Eleven proteins were detected by a proteomic analysis among them were adenosine triphosphate (ATP) synthase chain and two variants of enolase, described for the first time in CD patients [38]. The authors cited an Italian study describing not only a cytosolic form of alpha-enolase, but also the membrane variant of this enzyme recognized by autoantibodies [39]. The authors also suggested that the membrane-associated form probably could be a receptor and the binding of autoantibodies to its receptor could lead to cell damage. Thus, a new antigen was found and a novel possible mechanism of pathogenesis of CD was suggested [38]. In 2011, a group of Italian scientists explored the diagnostic and pathogenetic aspects of AAE Ab in patients with different inflammatory diseases. The studied population included: juvenile rheumatoid arthritis (n = 31), CD (n = 55), Crohn's disease (n = 59), hereditary periodic fever (n = 20), and periodic fever associated to aphthous, pharyngitis, and cervical adenopathies (PFAPA) (n = 28) compared with healthy blood donors (n = 80). AAE Ab (IgG and IgA) were detected by ELISA. Intriguingly, a low titer of AAE IgA and a quite elevated AAE IgG were detected in CD sera. This proposes that the activation of autoreactive B cells against ENO1 could be a systemic event and not just a local, mucosal one [40]. Three years later, Polish scientists studied AAE Abs from patients with CD (n = 31) compared with healthy controls (n = 6), using ELISA. The study demonstrated that CD patients had higher titers of AAE Ab, compared with the healthy subjects. At the same time, non adhering to gluten free diet (GFD) CD patients had higher titers of AAE Ab compared to the compliant subjects. The authors suggested that those antibodies might be a novel biomarker for enteric chronic inflammation among non-compliant CD patients [41]. In 2016, the same group studied the concentrations of anti-ganglioside M1 (anti-GM1) antibodies, NSE, interleukin 10 (IL-10) and their association with autonomic nervous system impairment in CD [42]. Sera from patients with CD (n = 34) and healthy controls (n = 34) were tested for antiendomysial antibodies by immunofluorescence and anti-tissue transglutaminase (anti-tTG), anti-GM1 antibodies, NSE and IL-10 by ELISA tests. No significant effect of CD on the average level of NSE concentrations was observed. More so, no significant correlation of the NSE concentration with either anti-GM1 antibodies, IL-10 and electrogastrography parameters were detected. According to the results of the study, anti-GM1 antibodies and IL-10 may be considered as markers of the nervous system impairment in the cases of CD. However, the role of NSE in the nervous system impairment in the course of CD could not be confirmed [42]. The final conclusion was that the CD intestinal chronic inflammation may be a reason for the autonomic nervous system impairment and development of neurologic disorders, whereby NSE is not directly involved.

## 4 Discussion

Neuro-specific enolase is not only heavily distributed on neurons, neuroendocrine, APUD and cancerous cells, but also exist in various autoimmune diseases including gastrointestinal inflammatory conditions like Crohn's and celiac diseases [22,37,38]. Its family member, ENO1 is much more abundant and have a ubiquitous distribution. It appears that both enolases are expressed at the intestinal levels, but their role in enteric diseases is far from being unraveled. The gut wall is heavily populated by the enteric nervous systems, localized in the sub-epithelial, inter-muscular or the glial cells [13], thus creating the base for the local NSE expression [22,37,38]. On the other hand, being a hyper-metabolic organ, not surprising is the fact that ENO1 populates the gut mucosa [40,41]. Presently, ENO1 and its specific antibodies, the AAbs, are reviewed in CD, in order to enlighten some aspects and suggest functional inter relationship between both. As mentioned above, ENO1 expression and AAbs titers are positively related to CD intestinal inflammatory activity [40,41]. Moreover, AAbs titers are increased in non-compliant patients, not adhering to gluten withdrawal [41]. It can be concluded that ENO1 inhabit the human gut, reflects intestinal inflammation and AAbs are gluten intake dependent. Table 1 summarizes potential shared aspects between CD and ENO1.

**Table 1:** Potential shared aspects between CD and ENO1.

Alpha-enolase	Celiac disease	References
Intestinal distribution	Classical enteric disease	[1,40,41]
AAE Abs are gluten dependent	All CD diagnostic autoantibodies are gluten dependent	[1,7,41,43,44]
Associated with cardiomyopathy	Dilated cardiomyopathy	[45,46]
Associated with cancer	A precancerous condition	[47,48]
Regulates α1+α2 Interferon	Interferon induced inflammation and intestinal damage	[49,50]
Regulated by iron	High incidence of iron deficiency	[[51], [52], [53]]
Stimulates dendritic cells	Gliadin peptides presenting cell	[54,55]
Enhances extra cellular matrix destruction	extra cellular matrix destruction in the enteric mucosa	[56,57]
Involved in pyruvate synthesis	Low pyruvate levels in CD mucosa.	[24,58]
Plasminogen receptor, activator of plasmin	Hypercoagulability	[8,57,59]
Molecular mimicry with rotavirus	Rotavirus is associated and rotavirus vaccination is protective	[[60], [61], [62]]
AAE Ab positivity in IBD, systemic and multiple sclerosis, Behçet's disease, rheumatoid arthritis, SLE, cryoglobulinemia and membranous nephropathy	All those autoimmune diseases are associated with CD	[12,[29], [30], [31], [32], [33],[63], [64], [65], [66], [67], [68], [69], [70]]
up-regulated in the brain of neurodegenerative diseases	Tissue transglutaminase and gluten are associated with neurodegenerative conditions	[[71], [72], [73]]
Expressed on peripheral lymphocytes (highest expression), erythrocytes, thrombocytes and serum	Intestinal origin of peripheral mature lymphocytes	[[9], [10], [11],^13^,^74^]
A substrate for posttranslational modification of transglutaminase	Tissue transglutaminase is the autoantigen	[75,76]
ENO1 is a cell wall protein responsible for transglutaminase activity in *Candida albicans*	*Candida albicans* is a potential inducer of celiac disease	[77,78]
Eno1 has TGase activity	Moonlighting Proteins at the Candidal Cell Surface	[79]

As shown in Table 1, multiple factors, mechanisms, pathways, co-morbidities and clinical presentations are shared between ENO1 and its corresponding AAE Ab and CD or with CD-associated autoimmune diseases. Intriguingly, it appears that ENO-1 exhibits a plethora of activities, which strongly depend on the enzyme extracellular and cellular localization [80]. Its sequestration on the cell surface [[71], [72], [73], [74], [75],^77^,^80^] enables ENO1 to react with numerous cell surface components and extracellular compartments' molecules. In parallel, the transglutaminase enzyme is not only a cell membrane protein, hence, it is secreted into the intercellular space. The potential opportunity for ENO1 to encounter and be modified by the tissue transglutaminase, as an acyl donor, is readily available. Both enzymes are ubiquitous, spanning numerous cells, tissue, organs and extracellular compartments, thus affecting a plethora of biological and pathological processes [24,36,71,81,82]. Notably, in an extensive proteomic survey, ENO-1 was found as the most differentially expressed protein in humans regardless of a cellular, tissue, organ types and pathological conditions [83]. The abilities of ENO-1 and tissue transglutaminase to conduct so many diverse processes are reflected by their involvement and contribution to a vast number of pathologies. Moreover, tissue transglutaminase is over activated in the intestinal inflamed sub-epithelial space in naïve CD [76,82,84]. Both enzymes are activated in inflammatory environment and CD is a classical enteric inflammatory condition. Broad range of inflammatory stimuli has been shown to stimulate expression of ENO-1 and to activate the local transglutaminase, including in mucosa of CD patients [[38], [39], [40],^76^,^80^,^82^]. Interestingly, enteric neuronal density contributes to the severity of intestinal inflammation [85] and NSE and ENO1 are expressed on neuronal cells including at the level of the CD mucosa [[37], [38], [39], [40], [41]]^.^ Moreover, mucosal neuroproliferation is a feature of CD [86], thus reinforcing the CD enteric inflammation-gut nervous system-enolases cross-associations.

Intriguingly, NSE is one of the marker antigens of the enterochromaffin cells located at the intestinal crypts [87]. Tumors derived from these cells, namely carcinoids are 15 times more common in patients with autoimmune Crohn's disease than in those without it [88]. Crohn's disease, CD and carcinoids are known for common biostructure of fecal microflora [89]. Notably, carcinoid morbidity associated with CD is well known [90,91]. Even in the absence of carcinoid in CD patients, the amount of enterochromaffin cells in the intestinal mucosa is much greater than in normal conditions [92,93], resulting in hyper serotoninemia in CD, just slightly less manifested than in carcinoids [94,95]. Serotonin can influence the interaction of T lymphocytes and dendritic cells, stimulating lymphocytes proliferation and gut inflammation [96,97]. Both availability and effects of those enterochromaffin cells product in gut are increased in CD [98]. It is possible to assume that autoantibodies to NSE in CD can be elicited as a result of protective reaction against hyperplasia and probable neoplasia of enterochromaffin cells. Hence, the anti-NSE autoantibodies, as a counter-regulating factor for expansion on enterochromaffin cells in CD, possibly can be protective for CD, regarding both inflammation and carcinoid risk. Interestingly, similar situation is observed in chronic autoimmune thyroiditis, were huge excess of Hürthle-Askanazy cells expressing plenty of NSE [99] may co-exist with increase in AAE Ab [35]. Moreover, hyperplasia of gut enterochromaffin cells is common not only in CD, but also in autoimmune thyroiditis [100].

Several potential modes of action can be suggested for the AAE Ab. In autoimmune conditions and enteric inflammatory diseases, AAE Ab could induce endothelial damage through the generation of immune complexes and the complement classical pathway activation, inhibiting the binding of plasminogen to ENO1, thus perturbating the intravascular and pericellular fibrinolytic system functions, and induction of cell death through the apoptotic process [36]. Finally, when cross-reactivity of food specific antibodies against ENO1 were explored, high to moderate reactivities to wheat, alpha gliadin 33mer, milk, soy and egg were detected. The highest cross-reactivity was between anti-wheat antibodies, while the CD associated anti-supra-molecule alpha gliadin 33mer reacted moderately to ENO [101]. The molecular mimicry between food specific antigens and tissue components based on the cross-reactivity of their corresponding antibodies associated with autoimmunity induction was recently reported [102,103]. Zooming on ENO1 and CD, the topic of specific anti food antibodies reacting to ENO1 in CD patients should further be explored. The fact that gluten might be involved in neurodegeneration [104] adds a new potential pathophysiological mechanism relaying the enolases to extraintestinal manifestations of CD.

## 5 Conclusions

Multiple shared aspects between tissue transglutaminase and ENO1 put the two offending enzymes as prime candidates to explore the ENO1-tissue transglutaminase cross-talks in CD induction and evolvement. Mucosal inflammatory induction, intestinal damage, immune dysregulation, posttranslational modification of naïve proteins, autoimmunogenesis, autoantibodies secretion and over all the entire pathogenesis are highly influenced by the two ubiquitous enzymes. Being the autoantigen, tissue transglutaminase is a game changer in CD, however the place of ENO1 is far from being elucidated. Is Eno1 a new kid on CD mucosal block? Is it the hen or the egg? The jury is still out.

## Supportive foundations

This work was supported by the grant of the Government of the 10.13039/501100009432Russian Federation for the state support of scientific research carried out under the supervision of leading scientists, agreement 14. W03.31.0009.

## CReDiT author statement

Aaron Lerner: Conceptualization, Data curation, Writing – original draft, Validation. Polina Sobolevkaia: Conceptualization, Data curation, Formal analysis, Writing – original draft. Leonid Churilov: Conceptualization, Funding acquisition, Project administration, Writing – review & editing, Supervision. Yehuda Shoenfeld: Conceptualization, Funding acquisition, Project administration, Writing – review & editing, Supervision.

## Declaration of competing interest

The authors declare that they have no known competing financial interests or personal relationships that could have appeared to influence the work reported in this paper.
